# P-563. Low level viremia is associated with viral non-suppression within 96 weeks among adolescents with perinatally acquired HIV: a prospective cohort study

**DOI:** 10.1093/ofid/ofae631.761

**Published:** 2025-01-29

**Authors:** Jose Luis Paredes, Danai Bemplidaki, Nyasha V Dzavakwa, Molly Chisenga, Suzanne Filteau, Celia L Gregson, Lackson Kasonka, Katharina Kranzer, Hildah Banda-Mabuda, Hilda Mujuru, Nicol Redzo, Tsitsi Bandason, Sarah Rowland-Jones, Ulrich E Schaible, Victoria Simms, Rashida Ferrand

**Affiliations:** Advocate Illinois Masonic Medical Center, chicago, Illinois; The Health Research Unit Zimbabwe, Biomedical Research and Training Institute, Harare, Zimbabwe, chicago, Illinois; The Health Research Unit Zimbabwe, Biomedical Research and Training Institute, Harare, Zimbabwe, chicago, Illinois; University Teaching Hospital, Lusaka, Zambia., Lusaka, Lusaka, Zambia; London School of Hygiene & Tropical Medicine, London, UK., London, England, United Kingdom; Musculoskeletal Research Unit, Bristol Medical School, University of Bristol, Bristol, UK., Bristol, England, United Kingdom; University Teaching Hospital, Lusaka, Zambia., Lusaka, Lusaka, Zambia; The Health Research Unit Zimbabwe, Biomedical Research and Training Institute, Harare, Zimbabwe, chicago, Illinois; University Teaching Hospital, Lusaka, Zambia., Lusaka, Lusaka, Zambia; Department of Paediatrics, University of Zimbabwe, Harare, Zimbabwe, Harare, Harare, Zimbabwe; The Health Research Unit Zimbabwe, Biomedical Research and Training Institute, Harare, Zimbabwe, chicago, Illinois; The Health Research Unit Zimbabwe, Biomedical Research and Training Institute, Harare, Zimbabwe, chicago, Illinois; Nuffield Department of Medicine, University of Oxford, UK, Bristol, England, United Kingdom; Research Center Borstel, Leibniz Lung Center, Borstel, Germany, Borstel, Niedersachsen, Germany; London School of Hygiene & Tropical Medicine, London, England, United Kingdom; The Health Research Unit Zimbabwe, Biomedical Research and Training Institute, Harare, Zimbabwe, chicago, Illinois

## Abstract

**Background:**

**V**iral suppression, defined as having a viral load (VL) < 1,000 cop/mL is used as the threshold for antiretroviral treatment (ART) changes in WHO guidelines. It is unclear whether those with low-level viremia (LLV) (VL 60-999 cop/mL) and undetectable (VL< 60) have different HIV-related outcomes. This study investigated factors associated with having viral non-suppression (≥1000 copies/ml) within 96 weeks among adolescents with perinatally-acquired HIV in Zimbabwe and Zambia with viral suppression (< 1000 copies/mL) at baseline.

**Methods:**

Longitudinal analysis of data from a trial using vitamin D and calcium supplementation on adolescents with perinatally-acquired HIV aged 11-19 years established on ART for > 6 months. Participants were enrolled from public-sector HIV-clinics in Harare and Lusaka and VL were measured at baseline, 48 weeks and 96 weeks. Those who completed follow-up until 96 weeks and were virally suppressed at baseline were included in this analysis. Enhanced adherence counselling was provided to those with viral non-suppression at 48 and 96 weeks. Multivariate Poisson regression was used for analysis of the association of viral-non suppression with VL at baseline, adjusting for age and sex a priori.

**Results:**

A total of 656 who had a VL< 1000 at baseline and complete follow-up were included in this analysis. Median age was 15.2 years, 54.6% were female, 90 (13.7%) had viral non-suppression within 96 weeks: 49 (7.5%) at week 48, and 43 (6.6%) at week 96. Viral non-suppression occurred among 18/54 (33.3%) of those who had LLV at baseline in comparison to 72/602 (12.0%) of those that were undetectable at baseline. Adjusting for age and sex, those with LLV were 2.3 times more likely to have viral non-suppression within 96 weeks (adjusted risk ratio 2.32, 95%CI 1.53-3.51) in comparison to those undetectable. In univariate analysis, country, age, sex, being enrolled in school and baseline ART regimen were not associated with having viral non-suppression within 96 weeks of follow up.

**Conclusion:**

Adolescents with LLV are at higher risk of viral non-suppression within 96 weeks than those with undetectable VL, with implications for HIV transmissibility, resistance to ART and HIV-related clinical outcomes.

**Disclosures:**

**All Authors**: No reported disclosures

